# Hybrid Forward Osmosis–Nanofiltration for Wastewater Reuse: System Design

**DOI:** 10.3390/membranes9050061

**Published:** 2019-05-06

**Authors:** Mattia Giagnorio, Francesco Ricceri, Marco Tagliabue, Luciano Zaninetta, Alberto Tiraferri

**Affiliations:** 1 Department of Environment, Land and Infrastructure Engineering (DIATI), Politecnico di Torino, Corso Duca degli Abruzzi 24, 10129 Turin, Italy; mattia.giagnorio@polito.it (M.G.); francesco.ricceri@polito.it (F.R.); 2 Decarbonisation and Environmental Laboratories, Eni S.p.A., Via F. Maritano 26, 20097 San Donato M.se, Italy; marco.tagliabue@eni.com; 3 Syndial S.p.A., Piazza M. Boldrini 1, 20097 San Donato M.se, Italy; luciano.zaninetta@syndial.it; 4 CleanWaterCenter@Polito, Politecnico di Torino, Corso Duca degli Abruzzi 24, 10129 Turin, Italy

**Keywords:** forward osmosis, nanofiltration, wastewater reuse, system design, fouling

## Abstract

The design of a hybrid forward osmosis–nanofiltration (FO–NF) system for the extraction of high-quality water from wastewater is presented here. Simulations were performed based on experimental results obtained in a previous study using real wastewater as the feed solution. A sensitivity analysis, conducted to evaluate the influence of different process parameters, showed that an optimum configuration can be designed with (i) an influent draw solution osmotic pressure equal to 15 bar and (ii) a ratio of influent draw solution to feed solution flow rate equal to 1.5:1. With this configuration, the simulations suggested that the overall FO–NF system can achieve up to 85% water recovery using Na_2_SO_4_ or MgCl_2_ as the draw solute. The modular configuration and the size of the NF stage, accommodating approximately 7000 m^2^ of active membrane area, was a function of the properties of the membranes selected to separate the draw solutes and water, while detailed simulations indicated that the size of the FO unit might be reduced by adopting a counter-current configuration. Experimental tests with samples of the relevant wastewater showed that Cl^−^- and Mg^2+^-based draw solutes would be associated with larger membrane fouling, possibly due to their interaction with the other substances present in the feed solution. However, the results suggest that fouling would not significantly decrease the performance of the designed system. This study contributes to the further evaluation and potential implementation of FO in water reuse systems.

## 1. Introduction

Forward osmosis (FO) is a promising technology for certain applications in water and wastewater treatment [1]. During the past two decades, studies have proposed FO as an alternative or complementary process with various objectives, such as desalination, treatment of liquid foods, or resource recovery from aqueous streams [2,3,4,5]. Owing to its low fouling propensity, the FO process is mainly recommended to treat complex waters characterized by large concentrations of organic components [6,7]. Several studies have been performed to evaluate draw solutions [2,8,9,10,11,12], to fabricate and characterize high-performance membranes [13,14], and to better control fouling [15,16]. When applied to extract high-quality water from a contaminated stream, FO needs to be coupled with a downstream separation process, which also regenerates the draw solution to be cycled back into the FO step. FO for waste/water treatment is arguably the most challenging configuration of this technology, in comparison with applications aiming at concentration or dilution of aqueous streams. Researchers and industry have not yet answered numerous questions regarding the feasibility and challenges of the entire system of coupling FO with downstream water recovery.

Researchers have analyzed forward osmosis performance in combination with other membrane filtration systems used for draw solution recovery, such as reverse osmosis or nanofiltration (NF) [17,18]. Nonconventional coupling technologies have also been proposed. For example, Xie et al. combined forward osmosis with membrane distillation for sewer mining [19], while Dutta et al. examined the possible application of eco-friendly draw solutes, such as ionic liquids, recoverable with low-grade thermal energy [20]. Recent studies also reported economic and environmental analyses of theoretical FO systems [21,22]. Most of the systems investigated so far were developed at laboratory scale, and the literature lacks studies to understand the implementations of full-scale systems. 

Operational parameters that highly influence the size of the system, its energy demands, and environmental impacts are, for example, the net driving force of the process, the inlet flow rate of the draw and feed solutions, and the recovery rate. Some modeling-based research was carried out to study the possible module design and to compare feasible configurations (e.g., cocurrent versus counter-current) [19,23,24,25,26]. These analyses are a step forward to understand the mass transfer mechanisms and they have not yet been developed to tackle the full scale of the potential treatment system. Only recently, Ali et al. developed a software tool to optimize the performance of a full-scale FO system based on the utilization of innovative spiral-wound elements [27]. This highly useful tool comprises the FO filtration as an individual treatment step. Nevertheless, it has been so far designed without accounting for the recovery process and without considerations related to performance reduction due to fouling.

This study presents a full-scale system design comprising both the forward osmosis step and a downstream nanofiltration stage, with the goal to extract high-quality water from wastewater. This design investigation is grounded on experimental results obtained with real wastewater at high recovery and was presented in our prior publication [28]. The previous experimental results are here integrated with a module-scale modeling investigation and a membrane fouling examination. Firstly, an analysis was performed to select the most important operational parameters, that is, the net driving forces, the draw solution flow rate in the FO step, and the overall recovery rate. The best design for the FO–NF system is thus presented, with an estimate of the overall membrane areas in the case of two potential draw solutes—MgCl_2_ and Na_2_SO_4_. The cocurrent and the counter-current configurations were briefly compared and, finally, membrane fouling was studied to assess its effects on water flux, causing possible deviations from the idealized simulations. 

## 2. Materials and Methods

### 2.1. FO Fouling Tests

#### 2.1.1. Lab Unit, Membranes, and Draw Solutes

Fouling tests were performed in forward osmosis with a lab-scale system. The system comprised a custom-made flat membrane cell, where a rectangular membrane sample of 21.28 cm^2^ (3.3 sq. inches) can be housed. Other characteristics of the FO lab unit were described in our previous publication [7]. The experiments were run in cocurrent mode, that is, feed and draw solutions entering and exiting from the same sides, with 1.5 L of initial feed and draw solutions. A constant (i) cross-flow rate of 0.75 L/min and (ii) temperature of 23 °C were maintained during the tests, while the flux across the membrane was computed by recording the change in volume of the feed solution in time through a computer-interfaced balance. The hydrodynamic conditions in the feed channels corresponded to a Reynolds number of roughly 2000. Flat-sheet polyamide thin-film composite FO membranes were used. Membranes were fabricated via interfacial polymerization of fully aromatic polyamide on top of a polysulfone-polyester support. The average transport characteristics of the FO membrane samples were obtained by following the protocol reported by Tiraferri et al. [7] and are reported in the Supporting Information (Table S1). 

Fouling tests were carried out with different draw solutions, with salts used individually: magnesium chloride, sodium sulfate, sodium chloride, magnesium sulfate, and calcium chloride, all purchased from Carlo Erba, Milan, Italy. They were run with a fixed initial osmotic pressure of 18.4 bar by using appropriate concentrations for each draw solution and calculated with OLI System Software. These concentrations corresponded to 0.262 M for MgCl_2_, 0.308 M for Na_2_SO_4_, 0.406 M for NaCl, 0.796 M for MgSO_4_, and 0.279 M for CaCl_2_.

#### 2.1.2. Wastewater Samples and Fouling Experiment Protocol

Wastewater samples were collected from a site located in northwest Italy (named “Site B” in our previous publication [29]) and used as the feed solution in all the fouling experiments. More precisely, this sample was a mix of civil and industrial wastewater with contaminated groundwater from a site being currently reclaimed. The wastewater composition is reported in Table 1. The total dissolved solids (TDS) concentration was 540 mg/L, with a resulting intrinsic osmotic pressure of roughly 0.5 bar. The duration of each fouling test was 24 h. During the first 8 h of operation, the nominal driving force was kept constant by additions of appropriate volumes of a stock draw solution and deionized water in the draw and feed reservoir, respectively, every 30 min. Afterwards, the filtration experiment was kept running without further adjustments. No physical or chemical cleaning was performed during the filtration tests, and experiments were carried out in duplicates or triplicates for each of the draw solutions. 

### 2.2. Modeling of the Forward Osmosis–Nanofiltration Fluxes

Simulations of FO water flux, *J_w_*, and reverse salt flux, *J_s_*, were performed by application of the following equations [7]:

(1) $$J_{w}=A\left\{\frac{\pi_{D}\exp\left(-\frac{J_{w}S}{D}\right)-\pi_{F}\exp\left(-\frac{J_{w}}{k}\right)}{1-\frac{B}{J_{w}}\left[\exp\left(\frac{J_{w}}{k}\right)-\exp\left(-\frac{J_{w}S}{D}\right)\right]}\right\}$$

(2) $$J_{s}=B\left\{\frac{C_{D}\exp\left(-\frac{J_{w}S}{D}\right)-C_{F}\exp\left(-\frac{J_{w}}{k}\right)}{1-\frac{B}{J_{w}}\left[\exp\left(\frac{J_{w}}{k}\right)-\exp\left(-\frac{J_{w}S}{D}\right)\right]}\right\}$$

where *A* is the active layer water permeance, while *S*, *B*, and *D* are the support layer structural parameter, the salt permeability coefficient, and the diffusion coefficient of the draw solute in water, respectively. These are parameters intrinsic to the membrane characteristics (*A* and *S*) or to the draw solute (*B* and *D*: Table 2 shows these values for each of the draw solutes involved in this study) [9,29]. In the equations, *C_D_*/*π_D_* and *C_F_*/*π_F_* represent the draw and feed concentrations/osmotic pressures, respectively, while the exponential terms account for both the internal ($-\frac{J_{w}S}{D})$ and external $\left(\frac{J_{w}}{k}\right)$ concentration polarizations. Finally, *k* represents the mass transfer coefficient at the active layer–solution interface, which is a function of the hydrodynamics in the membrane flow cell and was maintained at 68 L m^−2^ h^−1^ for all the simulations [29]. If the loss of draw solute due to reverse salt flux was negligible, Equations (1) and (2) could be simulated separately.

Simulations of the water flux across the membrane were performed (i) to isolate the effect of fouling on FO filtration (Case 1) and (ii) to perform a system-scale analysis of the forward osmosis–nanofiltration hybrid system (Case 2). The wastewater feed was simulated without considering any foulant concentrations and by simplifying the mixture of ionic species with an equivalent NaCl concentration providing the same overall osmotic pressure (i.e., 0.5 bar). 

#### 2.2.1. Case 1: Simulation of the Water Flux across the FO Membrane during Fouling Experiments

In Case 1, the predicted models provided information about the flux reduction due only to the loss of driving force during fouling experiments. This reduction can be compared with the observed trend of water flux, thus inferring information related to the effect of fouling. The initial osmotic pressure of the draw and feed solutions (DS/FS) were set equal to 18.4 and 0.5 bar, respectively, in accordance with the filtration experiments. The trend of the water flux was computed from the actual values of DS/FS osmotic pressures, known based on their dilution/concentration during the tests. 

#### 2.2.2. Case 2: System-Scale Analysis and Design of the Forward-Osmosis Nanofiltration System

In this case, Equations (1) and (2) were applied to perform a system-scale analysis and design of the FO stage of the system, treating the real secondary effluents flow rate of 76 m^3^/h. In a hypothetical FO plant working in cross-flow mode, the water flux across the membrane, whether spiral-wound or tubular, would vary along the length of the membrane module. In particular, the overall water flux, recovery, and required membrane area depends strongly on the (i) influent draw solution to feed solution flow rate ratio, DS:FS, (ii) the initial (influent) DS osmotic pressure (π_D_), and (iii) the module configuration (i.e., co- vs. counter-current). Therefore, simulations were firstly performed in cocurrent mode analyzing different combinations of DS:FS and influent draw solution osmotic pressure. For each simulation, the water flux was modeled through the discretization of the theoretical length of the membrane module, followed by calculation of the associated recovery and of the required membrane area. The system-scale analysis conducted to study the FO performance in counter-current mode was developed based on the results obtained in the previous cocurrent simulations by keeping the same boundary conditions, that is, the same DS:FS ratio and influent draw solution osmotic pressure. While Equations (1) and (2) were used to design the forward osmosis stage of the full-scale system, Wave software (DuPont) was instead employed to simulate the nanofiltration stage. Based on the results reported in our previous publication [30], the membrane system was designed to be operated with either sodium sulfate or magnesium chloride as the draw solute. 

## 3. Results and Discussion

### 3.1. Design of the Forward Osmosis–Nanofiltration Hybrid System

A previous laboratory investigation involving high-recovery tests suggested the potential to reclaim the contaminated water with the characteristics summarized in Table 1 using a hybrid forward osmosis–nanofiltration process [7]. Here, we proposed a design of this full-scale system as a step forward toward its implementation and as a strategy to further evaluate the feasibility of forward osmosis in general. The system was studied and optimized using a single salt draw solution of sodium sulfate or magnesium chloride. The first critical parameters to select in the design approach were (i) the nominal concentration and (ii) the flow rate of the draw solution entering the FO step. Ordinarily, the flow rate of the feed solution is fixed and related to the site characteristics. Therefore, the second parameter can be ultimately expressed as a ratio between the flow rates of the draw solution and the feed solution, referred to as the DS:FS ratio. Increasing both design parameters results in higher water fluxes and recovery values in the FO step. On the other hand, the NF regeneration of a large flow rate of draw solution to a large final solute concentration translates into high operational and capital costs of this stage. Clearly, there is an optimized configuration that ensues from this trade-off.

**Figure 1:**
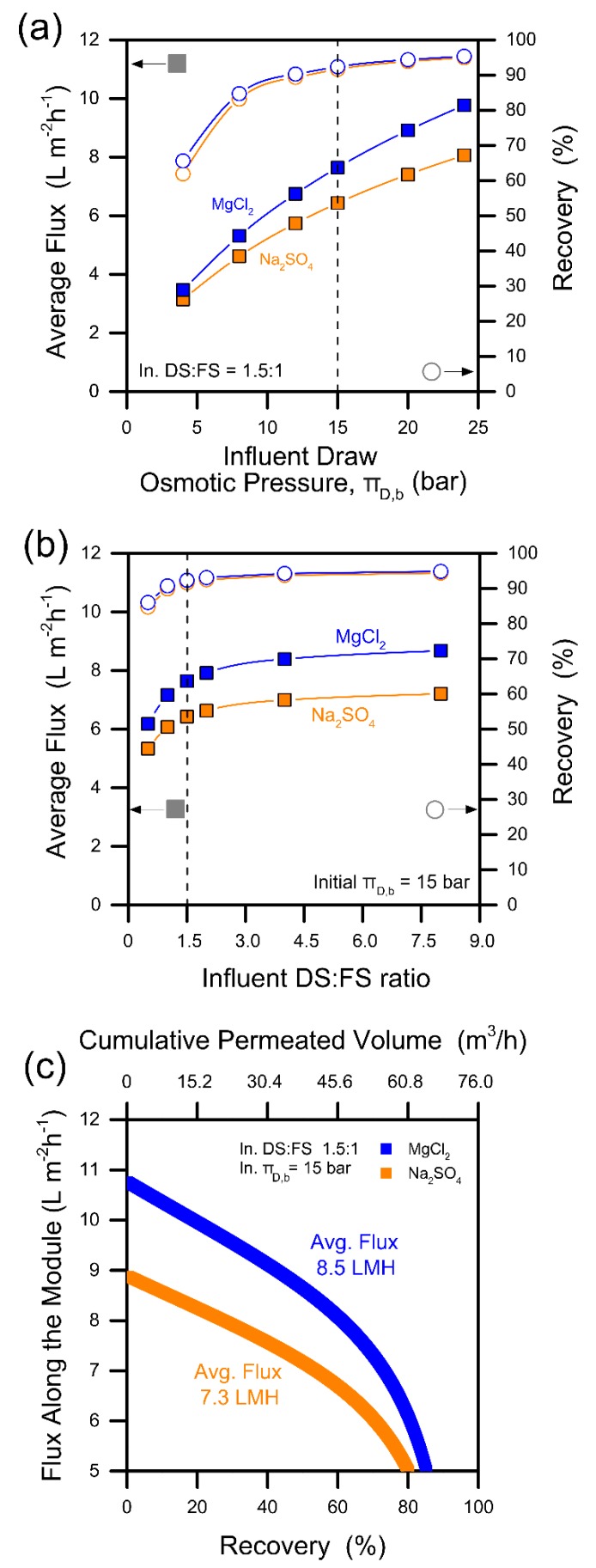
Choice of the forward osmosis (FO) operational parameters. The FO system was modeled in cocurrent mode. FO system recovery values (empty points) and related average modeled fluxes (full square points) as a function of (**a**) influent draw osmotic pressure and (**b**) influent ratio between the flow rates of the draw solution and the feed solution (DS:FS). Based on preliminary simulations presented in the Figure S1 of the Supporting Information, an influent DS osmotic pressure of 15 bar and an influent DS:FS ratio of 1.5:1 were considered, indicated by dashed lines. The curves in (**c**) are those modeled for the final FO system, considering the loss of driving force across the FO modules. Blue refers to MgCl_2_ and red to Na_2_SO_4_. Solid lines in (**a**,**b**) are intended as guide for the eyes only.

Module-scale simulations based on mass and volume balances and the membrane transport properties were thus performed to study the FO performance in terms of average flux and recovery rate by assuming a cocurrent FO configuration. The results are presented in Figure S1 of the Supporting Information. As expected, the FO performance was higher when increasing both the influent draw solution osmotic pressure and the DS:FS ratio. Nevertheless, the performance was not significantly improved above certain thresholds, implying that the optimization procedure would maximize the design parameters up until the magnitude of the first derivative of the curves in Figure S1 starts decreasing significantly. For this specific feed water, the best FO configuration appeared to be associated to an influent π_D_ of 15 bar and an influent DS:FS ratio of 1.5:1. These selections can be better rationalized in the graphs presented in Figure 1a,b. No significant benefits in terms of water productivity/recovery would be obtained from a DS:FS ratio larger than 1.5. An influent draw solution at an osmotic pressure > 15 bar would increase the average flux but without improving the overall recovery. In addition, Figure 1a,b show that higher average fluxes can be obtained in the FO unit with magnesium chloride as the draw solute. 

Based on the above selections, the local water flux along the hypothetical FO modules was calculated and is presented in Figure 1c. The flux is plotted as a function of the recovery rate and the cumulative permeated volume, both increasing along the modules. The simulations were halted for flux values lower than 5 LMH, as operating below this threshold would not be advantageous, resulting in a large increase of needed membrane area without a significant increase in the overall recovery rate. The recovery values were thus equal to 78% in the case of sodium sulfate and 85% with magnesium chloride as draw solutes. It is important to note that while these simulations took into account the transport-limiting concentration polarization phenomena, they did not consider the effect of fouling or membrane degradation in time. They were also based on a value of the mass transport coefficient representative of our lab-scale system, 68 LMH, which was certainly lower than that prevailing in real modules. As such, the external concentration polarizations would be reasonably lower in real operation and water fluxes would be higher.

**Figure 2:**
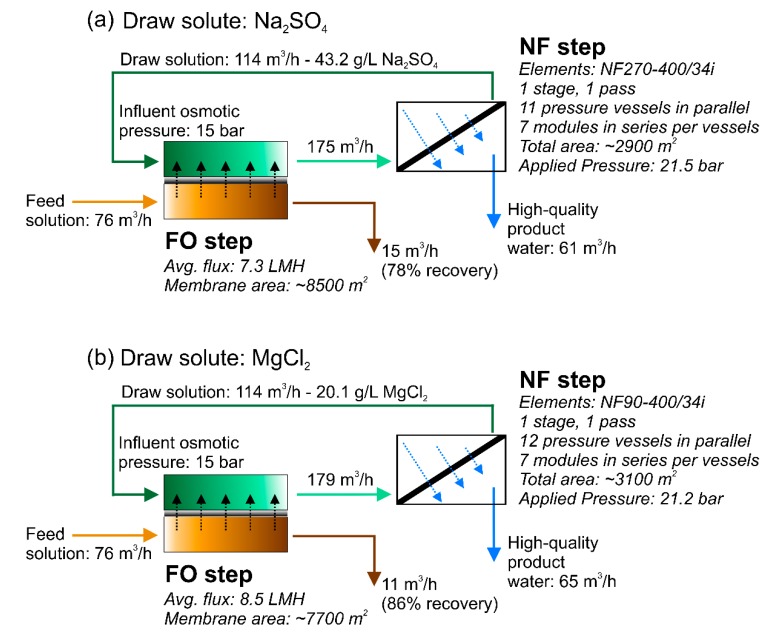
Configurations of the forward osmosis–nanofiltration hybrid system designed to treat real wastewater with (**a**) sodium sulfate and (**b**) magnesium chloride as the draw solute. The nanofiltration stage was designed to be operated with the NF270 and NF90 membranes, respectively [31].

Figure 2 shows the prospective entire configuration of the hybrid forward osmosis–nanofiltration system. Thanks to the higher average flux achievable in FO with MgCl_2_, this treatment step would require 10% less membrane area compared with the FO unit operated with Na_2_SO_4_. The nanofiltration regeneration stages were designed to recover the draw solution to achieve the desired influent osmotic pressure of 15 bar in the FO stage. The NF stage was significantly different depending on the type of DS. The NF90 and NF270 membranes (DuPont, Midland, MI, USA) were deployed to recover MgCl_2_ and Na_2_SO_4_, respectively. The characteristics of the NF membranes are reported in Table S2 of the Supporting Information and computed as described in our previous paper [31]. While the general configuration of the two NF options was similar, comprising both one stage and one pass and working at almost equivalent applied pressure (i.e., 21.5 and 21.2 bar for the NF270 and the NF90, respectively), the NF system designed to recover the sodium sulfate solution required less membrane area. This result was expected as NF270 has higher permeance compared with the NF90 [7]. 

Overall, both NF systems comprised seven modules in a series for each vessel. The simulations suggested that 12 parallel pressure vessels would be required in the NF system to recover magnesium chloride, while 11 vessels in the sodium sulfate regeneration system would be needed. The simulated quality of the permeate streams would be very high (not shown), with ion concentrations always below the stricter limits imposed for unrestricted irrigation. According to the calculations, the TDS value of the NF270 permeate would be 210 mg/L, while that of the NF90 permeate would be 90 mg/L. This is in accordance with our previous publication discussing experimental results at the laboratory scale [7].

In addition, simulations of the reverse salt fluxes in FO were performed to evaluate the loss of draw solutes in this step, which suggested the loss of less than 0.1% of the mass of draw solute for every hour of operation. If a conservative value of 98% for the overall observed solute rejection was considered for the NF step, roughly 0.9% of the mass of draw solute passed into the permeate every hour in this stage. These figures yielded a total needed replenishment of approximately 0.42/0.21 kg of Na_2_SO_4_/MgCl_2_ for each cubic meter of recycled draw solution or, equivalently, 48/24 kg of replenishment for every hour of operation, in comparison with a total amount of solutes of 4.9 and 2.4 tonn entering the FO unit every hour. 

### 3.2. Cocurrent versus Counter-Current Configuration

**Figure 3:**
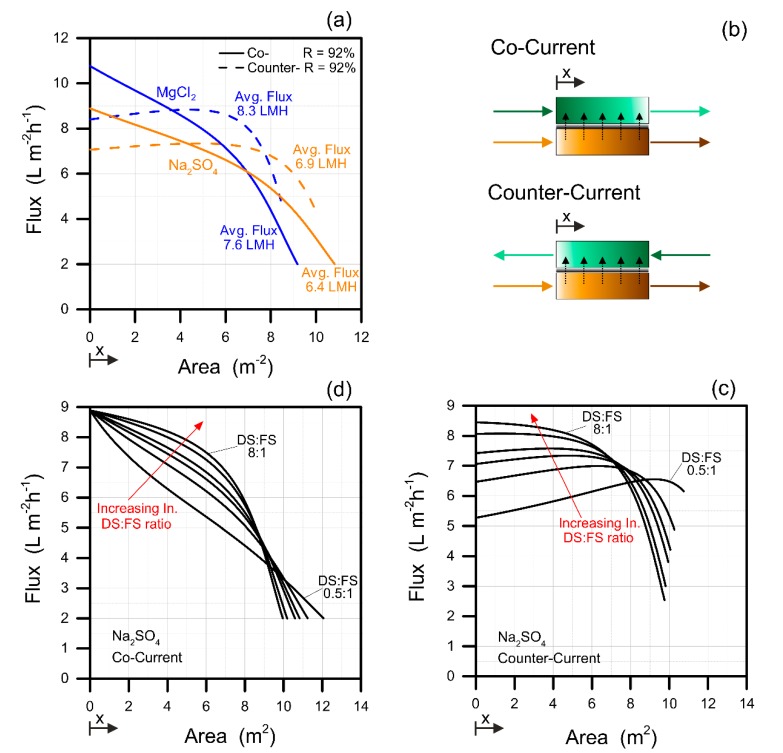
FO stage performance in co- vs. counter-current mode. (**a**) Water flux along the FO modules in the systems from Figure 2, for the (solid lines) cocurrent and (dashed lines) counter-current configurations. (**c**,**d**) Water fluxes along the modules at varying DS:FS ratios with influent π_D_ equal to 15 bar. The two configurations are schematically depicted in (**b**).

While module-scale FO simulations to understand the influence of operational parameters are more easily performed in cocurrent mode without the need of a complex numerical tool, counter-current mode is usually more practically advantageous. Therefore, the two modes were compared for some chosen configurations. In Figure 3a, the calculated water flux values are reported as a function of the membrane area required to run the relevant systems to treat the wastewater investigated in this study. These simulations were performed with the same FO boundary conditions, that is, considering the same DS:FS ratio equal to 1.5:1 and the same initial π_D_ equal to 15 bar, and imposing a recovery of 92%. Higher average fluxes can be achieved in FO with counter-current configuration with a consequent reduction of the membrane area. This result is in accordance with Deshmukh et al. [7]. 

Figure 3c,d present the simulations performed in cocurrent and counter-current modes by varying the influent DS:FS ratio. Although the counter-current configuration showed a generally more uniform flux along the membrane module, at one end of the system, specifically where the feed solution was highly concentrated, the flux was much lower than along the rest of the modules. This phenomenon was more pronounced at higher DS:FS ratios. On the other hand, the water flux decreased more linearly along the modules in cocurrent FO mode. Therefore, a lower degree of freedom appears to exist for the DS:FS parameter in the counter-current mode. 

These considerations have important implications for a hypothetical treatment plant. Firstly, the counter-current configuration does seem generally advantageous in terms of FO performance. Also, the fouling behavior is reasonably affected by the FO mode of operation. Previous studies suggested less severe fouling in the case of counter-current configuration compared with the cocurrent mode [23]. This result is sensible given that uniform fluxes more similar to the average value characterize the counter-current configuration. As fouling is somewhat proportional to flux via the permeation drag toward the membrane interface, high flux values at the influent end of the cocurrent configuration may result in exacerbated fouling at this end of the modules. Indeed, fouling is mostly related to the quality of the feed solution, and for this reason, pretreatment of the wastewater feed solution should be carefully considered to minimize the possible detrimental effect of fouling.

### 3.3. Fouling Propensity and Behavior

**Figure 4:**
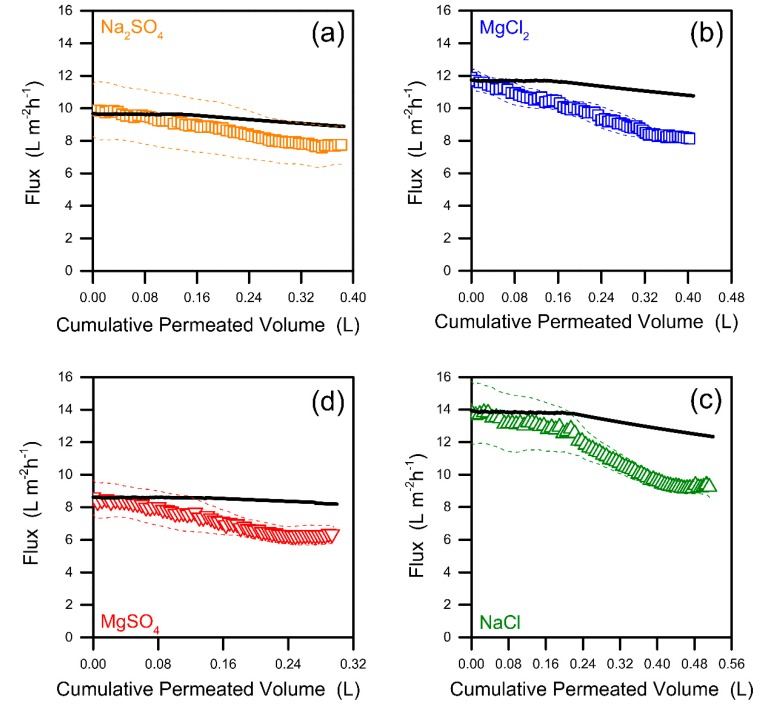
Results of the fouling experiments performed with (**a**) sodium sulfate, (**b**) magnesium chloride, (**c**) sodium chloride, and (**d**) magnesium sulfate as draw solutes, at initial draw osmotic pressure of 18.4 bar. The open points represent the average values of flux from duplicate or triplicate experiments. The dashed lines are the flux values with addition and subtraction of the standard deviation. The black lines depict the modeled fluxes, computed considering the sole reduction due to the loss of driving force in the batch tests, i.e., dilution of the draw and concentration of the feed solutions following water permeation.

All the simulations and the discussions presented so far were conducted without taking into account the detrimental effects of fouling. The FO flux decline due to fouling was evaluated experimentally using the wastewater of interest to assess the potential discrepancy with respect to the above calculations. The fouling results reported in Figure 4 refer to a very challenging condition in terms of hydrodynamics. As the Reynolds number was low, no spacers were used, and the initial water flux was always higher than the highest values simulated for each respective draw solute. Additional experiments with NaCl, MgSO_4_, and CaCl_2_ draw solutes were performed to understand the influence of ionic species on the fouling behavior. Data are presented for both experimental and modeled (flux reduction only due to loss in driving force) fluxes as a function of the cumulative permeated volumes. The contribution to flux decline due to fouling can be inferred from the difference between the two profiles. 

Before discussing the trends in flux, it should be highlighted that the model strongly agrees with the initial experimental flux values for all the draw solutions (see Figure 4). The model does not contain any adjustable parameter and is the result of the application of Equation (1) with the measured and known values of membrane transport properties and mass transfer coefficients. At fixed initial osmotic pressure, the FO performance varied significantly depending on the characteristics of the draw solute due to differences in *B* and *D* values [9,32]. NaCl produced the largest initial flux, mostly attributed to its high diffusion coefficient in water, which thwarts the effects of internal concentration polarization. 

In general, the fouling results suggest that the initial flux has some influence on the extent of flux decline, as a larger flux reduction was usually observed for larger values of initial flux. However, this is not the only factor at play, as MgSO_4_ caused more fouling-related flux reduction than Na_2_SO_4_, despite the latter producing a higher initial flux, and MgCl_2_ and NaCl resulted in similar fouling despite the tests comprising NaCl starting from significantly larger values of initial flux. Overall, the results suggest that chloride ions are associated with more flux decline compared with sulfate ions. This can be associated with the greater tendency of chloride to diffuse into the feed solution, thus facilitating the formation of a denser cake [33]. Also, magnesium is much more prone to produce flux decline than sodium. This is also in accordance with literature reports, discussing the role of Mg^2+^ in increasing the attachment of organic compounds to the membrane [34,35]. Multivalent cations are known to enhance the membrane fouling propensity due to their ability to interact with negatively charged organic substances [36]. A definite proof of this phenomenon was clearly visible for tests comprising CaCl_2_ as the draw solute, associated with very large flux declines (>50%), as presented in Figure S2 of the Supporting Information [37]. 

Another interesting observation from the fouling tests is related to the fact that a near stable flux was achieved around the end of the experiments performed with three out of the four draw solutes, suggesting the possibility of the existence of a critical, sustainable, or threshold flux, as suggested by previous authors [38,39,40,41,42]. Regardless of the debated mechanism underlying this phenomenon, the stabilization of flux suggests that there may be a limitation factor that slows down or nearly prevents further fouling after a certain point. This phenomenon may be related to a constraint in building up the foulant cake layer above a certain thickness, or it may be associated with kinetics considerations of foulant deposition onto a predeposited foulant layer due to foulant–foulant interactions. This observation is quite noteworthy, implying that the water flux may be trusted not to cross below a threshold value, which is most likely related to the complex system consisting of the membrane, the hydrodynamics, the draw solute, and the feed composition. Based on our results, the minimum flux with Na_2_SO_4_ may be only about 10% lower than the modeled flux (Figure 4a). Overall, the effect of fouling was not overly harsh, also considering that simple physical cleanings allowed the recovery of the near totality of the initial flux in almost all the cases (not shown) [43]. To conclude, the fluxes and the recovery rates discussed in Section 3.1 and Section 3.2 and presented in Figure 1, Figure 2 and Figure 3 may be reasonably utilized to predict those obtained in appropriately run and well-maintained real systems, considering a safety factor of >0.9 due to fouling effects. Certainly, more research should be conducted with real samples and using actual FO modules to approximate their behavior at a large scale. 

## 4. Conclusions

The system design and fouling propensity of a hybrid forward osmosis–nanofiltration system for the production of high-quality water from a secondary wastewater effluent with osmotic pressure of 0.5 bar was discussed in this manuscript. The sensitivity analysis performed on the forward osmosis filtration step showed the importance of (i) the nominal concentration of the draw solution and (ii) its relative flow rate in the process performance. An optimum FO configuration existed for this specific case, associated with an influent DS osmotic pressure of 15 bar and a DS:FS of 1.5:1. Overall, 80%/85% recovery can be achieved by the utilization of Na_2_SO_4_/MgCl_2_ as draw solutes, with a nanofiltration recovery step designed to work with either the NF270 or the NF90 membrane modules. 

To reduce the membrane area in the FO stage, a counter-current configuration should be adopted. Finally, fouling in the forward osmosis step was minor and mostly reversible. Fouling effects would be enhanced by using chloride- and magnesium-based draw solutes. Given the mild nature of fouling, all the idealized simulations performed for the FO unit, which did not include fouling effects, may be assumed to be valid for a well-maintained real system. A conservative strategy would include the adoption of a safety factor roughly equal to 0.9 to estimate average fluxes and recovery ratios from simulated values.

## Figures and Tables

**Table 1:** Composition of the wastewater from the location of interest.

Parameter	Value
Dissolved organic carbon (mg/L)	22
pH	8.0
Cl^‒^ (mg/L)	100
F^‒^ (mg/L)	0.1
PO_4_^3‒^ (mg/L)	1.5
NO_3_^‒^ (mg/L)	2.2
SO_4_^2‒^ (mg/L)	250
N-NH_4_ (mg/L)	0.004
Ca^2+^ (mg/L)	31
Mg^2+^ (mg/L)	13
K^+^ (mg/L)	15
Na^+^ (mg/L)	120
Al (µg/L)	70
As (µg/L)	4.2
Cr (µg/L)	1.3
Fe (µg/L)	16
Ni (µg/L)	3.7
Conductivity (µS/cm)	1100
Total dissolved solids (TDS) (mg/L)	540
Osmotic pressure (bar)	0.5

**Table 2:** *B* and *D* of the draw solutes.

Draw Solute	*B* (LMH)	*D* (m^2^/s)
Na_2_SO_4_	0.06	7.6 × 10^−10^
MgCl_2_	0.07	1.1 × 10^−9^
NaCl	0.94	1.5 × 10^−9^
MgSO_4_	0.39	4.3 × 10^−10^
CaCl_2_	0.16	1.1 × 10^−9^

## Supplementary Materials

The following are available online at https://www.mdpi.com/2077-0375/9/5/61/s1, Figure S1: Preliminary simulations performed to select the forward osmosis operational parameters, Figure S2: Results of the fouling experiments performed with calcium chloride as draw solution, at an initial osmotic pressure of 18.4 bar, Table S1: Transport properties of the forward osmosis membrane, Table S2: Characteristics of the nanofiltration membranes selected for the draw solution regeneration step.
