# Supplementary material for: Hybrid Forward Osmosis–Nanofiltration for Wastewater Reuse: System Design
Source: Membranes (Basel). 2019 May 6;9(5):61. doi: 10.3390/membranes9050061 (PMC6572449; doi:10.3390/membranes9050061)
Supplement: Supplementary file 1 [file membranes-09-00061-s001.docx]

Supporting Information: Hybrid Forward Osmosis–Nanofiltration for Wastewater Reuse: System Design

Mattia Giagnorio ^1,†^, Francesco Ricceri ^1,†^, Marco Tagliabue ^2^, Luciano Zaninetta ^3^, Alberto Tiraferri ^1,4,^*

^1^ Department of Environment, Land and Infrastructure Engineering (DIATI), Politecnico di Torino, Corso Duca degli Abruzzi 24, 10129 Turin, Italy; mattia.giagnorio@polito.it (M.G.); francesco.ricceri@polito.it (F.R.)

^2^ Decarbonisation and Environmental Laboratories, Eni S.p.A., Via F. Maritano 26, 20097 San Donato M.se, Italy; marco.tagliabue@eni.com

^3^ Syndial S.p.A., Piazza M. Boldrini 1, 20097 San Donato M.se, Italy; luciano.zaninetta@syndial.it

^4^ CleanWaterCenter@Polito, Politecnico di Torino, Corso Duca degli Abruzzi 24, 10129 Turin, Italy

^†^ The two authors contributed equally to this manuscript

***** Correspondence: alberto.tiraferri@polito.it

Received: 12 April 2019; Accepted: 2 May 2019; Published: date

**Table S1.** Transport properties of the forward osmosis membrane. The parameter *A* represents the active layer water permeance, *B* the salt permeability coefficient for sodium chloride (used for membrane characterization), and *S* the support layer structural parameter.

| ***A* (LMH/bar)** | ***B*_NaCl_ (LMH)** | ***S* (μm)** |
| --- | --- | --- |
| 2.74 ± 0.5 | 0.94 ± 0.25 | 427 ± 19 |

**Figure S1.** Preliminary simulations performed to select the forward osmosis operational parameters. The FO system was modelled in co-current mode. FO system recovery rates (squares) and average water fluxes (triangles), presented as a function of (**a** and **d**) influent DS:FS ratio and (**b** and **c**) influent draw osmotic pressure. The results are presented for sodium sulfate as draw solute. Solid lines are intended as guide for the eyes only.

**Table S2.** Characteristics of the nanofiltration membranes selected for the draw solution regeneration step. Solute rejection measured at a feed concentration of 30 mM and an applied pressure of 100 psi (6.9 bar).

| **Membrane** | **NF270** | **NF90** |
| --- | --- | --- |
| Active layer material | Polyamide | Polyamide |
| Water permeance (LMH/bar) | 18.8 | 6.9 |
| MgCl_2_ rejection (%) | 80.8 | 99.3 |
| Na_2_SO_4_ rejection (%) | 98.7 | >99.5 |

**Figure S2.** Results of the fouling experiments performed with calcium chloride as draw solution, at an initial osmotic pressure of 18.4 bar. The open points represent the average values of flux from duplicate experiments. The dashed lines are the flux values with addition and subtraction of the standard deviation. The black lines depict the modelled fluxes, computed considering the sole reduction due to the loss of driving force, i.e., dilution of the draw and concentration of the feed solutions following water permeation.
